# In-Process Recycling of 35% Glass Fiber-Reinforced Polyamide 6,6 Runners: Effects on Thermomechanical Properties and Viability for Diesel Injector Socket Production

**DOI:** 10.3390/polym17192569

**Published:** 2025-09-23

**Authors:** Elif Sahiner, Yasin Altin

**Affiliations:** 1 Department of Polymer Materials Engineering, Faculty of Engineering and Natural Sciences, Bursa Technical University, 16310 Bursa, Turkey; 2 Central Research Laboratory, Bursa Technical University, 16310 Bursa, Turkey

**Keywords:** circular economy, glass fiber composites, in-process reuse, mechanical recycling, Polyamide 6,6, sustainable manufacturing

## Abstract

Significant pre-consumer waste in the form of runners is generated during the injection molding of high-performance automotive components, representing both a substantial economic loss and an environmental burden. This study therefore comprehensively evaluated the mechanical recycling of pre-consumer 35% glass fiber-reinforced Polyamide 6,6 (%35GF-PA66) runners for in-process reuse in diesel injector socket production. The effects of blending recycled polymer (RP) at 2.5%, 5%, 10%, and 15% by weight and up to 10 recycling cycles with 15 wt.% RP on the thermal, mechanical, and morphological properties were investigated. Tensile strength slightly decreased (~3% at 10% RP) compared to virgin material, while elongation at break increased with higher RP content. Multiple recycling cycles had minimal impact on tensile strength, and the heat deflection temperature (HDT) remained nearly constant (~0.7 °C variation after 10 cycles, within experimental uncertainty). The melt flow index (MFI) increased significantly with successive recycling cycles, indicating molecular weight reduction due to thermomechanical degradation. DSC analysis confirmed stable melting and crystallization temperatures (variation < 1 °C), suggesting preserved crystalline structure. SEM analysis revealed increased void formation at the fiber–matrix interface and fiber attrition with successive recycling, correlating with reduced flexural properties. In-process recycling of %35GF-PA66 runners is viable, particularly at ≤15% RP and fewer cycles, offering significant cost savings (e.g., ~EUR 344,000 annually for a large producer) and environmental benefits.

## 1. Introduction

The automotive industry is continually striving for enhanced fuel efficiency, reduced emissions, and improved vehicle performance, leading to an increasing adoption of lightweight and high-performance materials [1,2,3]. Among these, fiber-reinforced polymers (FRPs), particularly Polyamide 6,6 reinforced with short glass fibers (PA66-GF), have become indispensable for manufacturing a wide array of structural and functional components due to their excellent balance of mechanical strength, stiffness, thermal stability, and processability [4,5,6,7,8,9]. PA66-GF composites, such as %35GF-PA66 (35 wt.% glass fiber), are extensively utilized for demanding under-the-hood applications, including critical parts like diesel injector sockets, where operational reliability under thermomechanical stress is paramount.

The predominant manufacturing process for such intricate PA66-GF components is injection molding, prized for its high production rates and ability to produce complex geometries [10]. However, a significant drawback of injection molding is the inevitable generation of pre-consumer (in-plant) waste in the form of runners, sprues, and defective parts. For instance, a major tier-1 automotive supplier can generate approximately 80 tons of such plastic waste annually from a single process line [11], representing not only a substantial economic loss due to discarded high-value material but also a considerable environmental burden. For complex, high-performance components, the runner system can account for a significant portion of the total shot weight: for the specific component in this study, the runner system constitutes 13% of the total shot weight, making this waste stream particularly substantial. This situation conflicts with global sustainability goals, such as the circular economy, the EU Green Deal, and national zero-waste initiatives, which promote resource efficiency and waste minimization [12,13]. The potential annual cost saving from effectively recycling this runner waste, estimated at EUR 344,000 for a large producer [11], further underscores the strong economic incentive for developing robust in-process recycling strategies.

Mechanical recycling, which typically involves grinding the waste material and re-introducing it into the production stream, often blended with virgin polymer, is the most straightforward and economically viable approach for thermoplastic composites [14,15,16]. However, the reprocessing of fiber-reinforced thermoplastics is not without challenges, with the final properties of the recycled composite being highly dependent on several interconnected factors. The primary degradation mechanisms have been specifically documented for PA66-GF composites.

A primary issue is (i) fiber attrition, resulting in a reduction of fiber length and aspect ratio [17,18]. This phenomenon has been well documented specifically for PA66-GF by Eriksson et al. [17], who showed significant reductions in average fiber length and a corresponding decline in tensile and impact strength after multiple processing cycles. However, the relationship between fiber length and mechanical properties is not always straightforward. For instance, in a foundational study on glass fiber-reinforced polypropylene, Thomason and Vlug demonstrated that while strength is sensitive to fiber length, the composite’s stiffness (modulus) can be virtually independent of fiber length above a certain critical minimum (~0.5 mm) [18]. Concurrently, (ii) the polymer matrix itself undergoes degradation, which alters its rheological and mechanical performance. This is often indicated by changes in melt viscosity, a key finding in the work of Gültürk and Berber [19], who highlighted significant changes in MFI and morphology with repeated recycling of PA66-GF. Similarly, Bernasconi et al. [20] linked these degradation effects to performance, observing a decrease in elastic modulus, tensile strength, and fatigue behavior with increasing recycled content.

Beyond the material’s intrinsic changes, (iii) fiber distribution and (iv) the specific recycling methods used are also critical. The work by Thomason and Vlug also revealed that high concentrations of long fibers can lead to packing problems and increased void content, which in turn reduces stiffness [18]. This aligns with findings from Ghabezi et al. [21] on recycled HDPE composites, where fiber agglomeration at high loadings created stress concentration points. The processing route itself is equally important. Akonda et al. [22] showed that converting recycled carbon fibers into aligned yarns before manufacturing composites was crucial for achieving acceptable mechanical properties. The complexity increases further when dealing with multi-component systems; Pegoretti et al. [23], for example, investigated ternary composites of recycled PET, glass fibers, and impact modifiers, highlighting the dominating effect of fiber reinforcement on long-term properties like creep. In some cases, reprocessing can even be beneficial. The “upcycling” concept, demonstrated by Sam-Daliri et al. [24], shows that using recycled 3D-printed parts as feedstock for injection molding can improve the final component’s properties by enhancing fiber distribution and reducing porosity.

Despite these varied degradation mechanisms, recycling often remains a viable strategy, as confirmed by Pietroluongo et al. [16], who showed that even end-of-life PA66-GF components, while degraded, could retain characteristics acceptable for less demanding applications.

While these studies provide valuable insights into the general recyclability of PA66-GF systems, there remains a specific need to understand the implications of in-process recycling of pre-consumer runner waste directly back into the production of the same critical component. The performance requirements for diesel injector sockets are stringent, and any degradation in material properties could have significant safety and operational consequences, potentially affecting engine reliability and efficiency. Furthermore, the cumulative effect of multiple recycling generations on material specifically sourced from runners, which experience a slightly different thermal history than the main part, needs detailed investigation. This study therefore aimed to comprehensively evaluate this specific industrial scenario.

This study aimed to comprehensively evaluate the feasibility of reusing mechanically recycled %35GF-PA66 runner waste from diesel injector socket production directly within the same manufacturing line. We systematically investigated the effects of: (a) incorporating varying weight percentages of recycled material (RP) with virgin %35GF-PA66, and (b) subjecting a fixed blend of recycled and virgin material to multiple (up to ten) reprocessing cycles. The impact of these recycling strategies on the critical mechanical properties (tensile, flexural, impact), thermal characteristics (HDT, DSC, TGA), melt flow behavior (MFI), and morphology (SEM) of the resulting composites was thoroughly assessed. The overarching goal was to determine the optimal conditions and limitations for the in-process reuse of this high-value waste stream, thereby contributing to more sustainable and cost-effective manufacturing practices in the automotive sector without compromising component integrity.

## 2. Materials and Methods

### 2.1. Materials

The virgin material used was Polyamide 6,6 reinforced with 35 wt.% short glass fibers (%35GF-PA66), commercially available as RADILON A RV350LW 358 BK (RadiciGroup, Bergamo, Italy). The pre-consumer waste consisted of runners generated during the injection molding of diesel injector sockets, provided by Bosch Sanayi ve Tic. A.Ş. (Bursa, Turkey).

### 2.2. Recycling and Specimen Preparation

The virgin material used was Polyamide 6,6 reinforced with 35 wt.% short glass fibers (%35GF-PA66), commercially available as Radilon A RV350LW 358 BK (RadiciGroup, Bergamo, Italy). The pre-consumer waste consisted of runners generated during the injection molding of diesel injector sockets at a Tier-1 automotive supplier manufacturing facility.

The %35GF-PA66 runners were first mechanically ground into granules using a Tmax WSGJ granulator (Wensui Plastics Machinery Group, Guangzhou, China).

Two sets of experiments were conducted, as follows.

1. *Effect of Recycled Content:* Ground recycled polymer (RP) was dry-blended with virgin %35GF-PA66 pellets at different weight percentages: 0% (virgin), 2.5%, 5%, 10%, and 15% RP.
2. *Effect of Multiple Recycling Cycles:* Based on initial performance, a fixed blend of 15% RP was chosen. Test specimens were molded, and the runners from this process were ground and re-blended with 85% virgin material to maintain 15% RP content for the next cycle. This process was repeated for up to 10 cycles.

All materials were dried at 70 °C for 5 h in a hot air oven prior to injection molding. Standard test specimens (e.g., tensile bars in accordance with ISO 527-2 [25]) were produced using an Engel Spex Victory 80 injection molding machine (ENGEL Austria GmbH, Schwertberg, Austria). Key injection molding parameters are detailed in Table 1.

### 2.3. Characterization

**Mechanical Testing**

- At least five specimens were tested for each condition for mechanical tests.
- Tensile tests were performed in accordance with ISO 527-2 [25] using a Shimadzu AGS-X universal testing machine (Shimadzu Corporation, Kyoto, Japan) at a crosshead speed of 5 mm/min. Type 1A multipurpose test specimens (170 mm × 10 mm × 4 mm) were used. Tensile strength, elongation at break, and Young’s modulus were determined.
- Three-point bending tests were conducted in accordance with ISO 14125 [26] on the same machine at 5 mm/min. Rectangular specimens with dimensions of 80 mm × 10 mm × 4 mm were tested. Flexural strength, flexural strain, and flexural modulus were recorded.
- Charpy impact tests were performed on unnotched specimens in accordance with ISO 179-1 [27] using an Instron CEAST 9050 impact tester (Instron, Norwood, MA, USA).

**Thermal Analysis**

- Heat deflection temperature (HDT) was measured in accordance with ISO 75-2 (method A, 1.80 MPa load) [28] using an Instron/Ceast HV3 apparatus (Instron, Norwood, MA, USA).
- Differential scanning calorimetry (DSC) was performed using a TA Instruments DSC250 (TA Instruments, New Castle, DE, USA) in accordance with ISO 11357-1 [29]. Samples (5–10 mg) were heated from 20 °C to 320 °C at 20 °C/min, held for 5 min, cooled to 0 °C at 20 °C/min, and then reheated to 320 °C at 20 °C/min under a nitrogen atmosphere. Melting (T_m_) and crystallization (T_c_) temperatures and enthalpies were determined.
- Thermogravimetric analysis (TGA) was carried out using a TA Instruments Discovery SDT 650 (TA Instruments, New Castle, DE, USA) in accordance with ISO 11358-1 [30]. Samples were heated from room temperature to 600 °C at 20 °C/min under nitrogen, then to 900 °C under oxygen atmosphere to determine degradation temperatures and residual ash content.
- Ash content was also determined separately in accordance with ISO 3451:2019 (method A) [31] by heating samples at 950 °C for 3 h.

**Melt Flow Index (MFI):** MFI was measured in accordance with ISO 1133 [32] using an Instron MFI tester (Instron, Norwood, MA, USA) at 280 °C with a 5 kg load.

**Morphological Analysis:** Fracture surfaces of tensile specimens and ash residues were examined using a Zeiss-Gemini 300 field-emission scanning electron microscope (FE-SEM) (Carl Zeiss AG, Oberkochen, Germany) at an accelerating voltage of 5 kV. Samples were sputter-coated with a thin layer of gold before observation.

## 3. Results and Discussion

### 3.1. Effect of Recycled Content (%RP) on Material Properties

The initial phase of this study focused on understanding how the incorporation of varying weight percentages of recycled polymer (RP) from %35GF-PA66 runners affects the primary mechanical properties of the composite after a single reprocessing cycle. For this purpose, tensile and three-point bending tests were conducted on specimens prepared with 0% (virgin), 2.5%, 5%, 10%, and 15% RP.

#### 3.1.1. Tensile Properties

**Figure 1:**
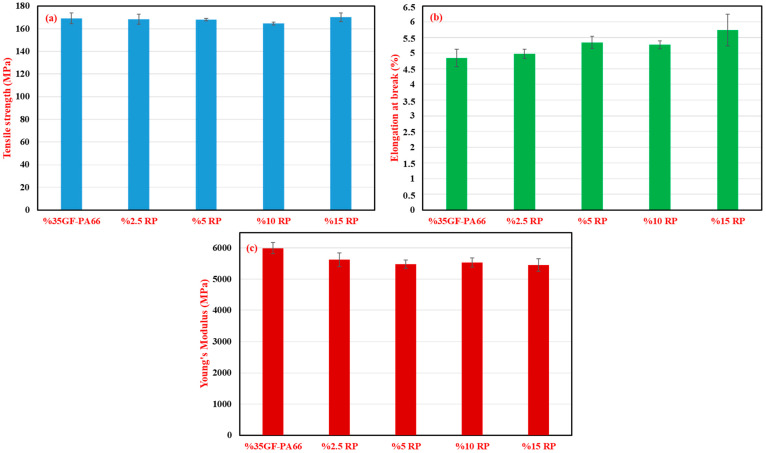
Effect of recycled polymer (RP) content (0–15%) on the tensile properties of %35GF-PA66 composites: (**a**) tensile strength (MPa), (**b**) elongation at break (%), and (**c**) Young’s modulus (MPa).

The results of the tensile tests, including tensile strength, elongation at break, and Young’s modulus, are graphically presented in Figure 1. Virgin %35GF-PA66 exhibited a tensile strength of 169.23 ± 4.70 MPa. The introduction of recycled material led to minor variations in tensile strength: 168.48 ± 4.40 MPa for 2.5% RP and 168.10 ± 1.10 MPa for 5% RP. A slight decrease was observed at 10% RP, with a tensile strength of 164.23 ± 1.28 MPa, representing an approximate 3% reduction compared to the virgin material. Interestingly, the blend containing 15% RP showed a tensile strength of 170.10 ± 3.70 MPa, which is comparable to and even slightly higher than the virgin material, though this could be within the bounds of experimental variability.

Elongation at break (Figure 1b) showed a general increasing trend with higher RP content, starting from 4.84 ± 0.26% for the virgin %35GF-PA66 and reaching 5.74 ± 0.29% for the 15% RP blend. This suggests a slight increase in the material’s ductility, potentially due to the thermomechanical degradation during reprocessing, which causes chain scission in the PA66 matrix. The resulting shorter polymer chains increase segmental mobility, acting as a minor internal plasticizer [20,33,34].

Young’s modulus (Figure 1c) displayed a tendency to decrease with the addition of RP. The virgin material had a modulus of 5995.05 ± 148.28 MPa, which reduced to a range of approximately 5530 MPa (for 10% RP) to 5670 MPa (for 15% RP) for the blends. This reduction in stiffness is consistent with the expected effects of fiber attrition during the grinding of runners and subsequent reprocessing. The observed decrease in Young’s modulus is primarily attributed to fiber attrition. Shorter fibers are less effective in transferring stress, which directly impacts the stiffness of the composite. Conversely, the tensile strength remained relatively stable. This can be explained by the complex nature of tensile failure, which is influenced by both fiber reinforcement and matrix properties. While fiber shortening may have a slight negative impact, concurrent polymer degradation (chain scission) can lead to a minor plasticizing effect, slightly increasing the matrix’s ductility. These two competing effects appear to balance each other, resulting in a negligible net change in the ultimate tensile strength within the observed experimental variability [17,18].

#### 3.1.2. Flexural Properties

**Figure 2:**
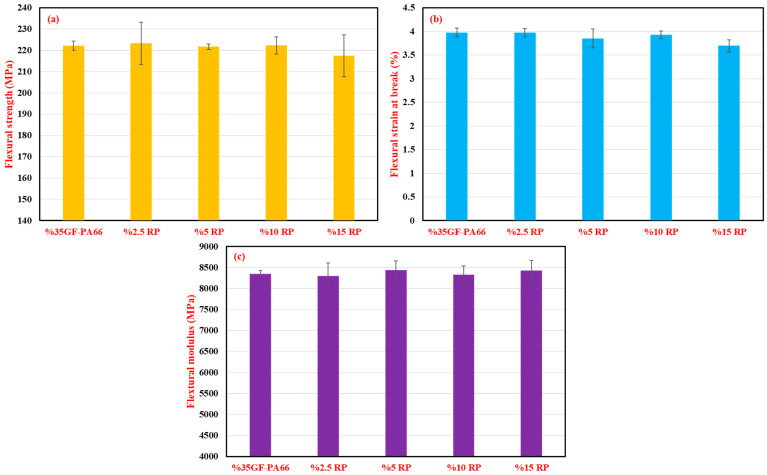
Effect of recycled polymer (RP) content (0–15 wt.%) on the flexural properties of 35% glass fiber-reinforced PA66 composites: (**a**) flexural strength (MPa), (**b**) flexural strain at break (%), and (**c**) flexural modulus (MPa).

The flexural properties, including flexural strength, flexural strain at break, and flexural modulus, for different RP contents are illustrated in Figure 2. Virgin %35GF-PA66 showed a flexural strength of 222.22 ± 2.22 MPa. Similarly to tensile strength, the flexural strength of the blends varied slightly: 223.32 ± 9.96 MPa (2.5% RP), 221.71 ± 1.33 MPa (5% RP), 222.30 ± 4.02 MPa (10% RP), and a slight decrease to 217.52 ± 9.80 MPa for 15% RP.

Flexural strain at break (Figure 2b) ranged from 3.98 ± 0.10% for virgin material to 3.70 ± 0.13% for 15% RP, showing a slight decreasing trend.

The flexural modulus (Figure 2c) for virgin %35GF-PA66 was 8348.64 ± 77.18 MPa. The blends with RP generally exhibited comparable or slightly lower flexural moduli, for instance, 8297.07 ± 307.31 MPa for 2.5% RP and 8431.65 ± 234.20 MPa for 15% RP. The variations in flexural properties can also be attributed to the combined effects of fiber length reduction and potential minor changes in the matrix due to the incorporation of reprocessed material [33,34,35].

Based on these initial mechanical property evaluations, the blend containing 15% RP was selected for further investigation involving multiple reprocessing cycles. This selection was made considering that even at this relatively higher RP content, the primary mechanical properties (tensile and flexural strength) remained largely comparable to the virgin material, providing a suitable baseline to study the cumulative effects of repeated recycling. The subsequent sections detail the impact of multiple recycling cycles on a broader range of properties, including thermal, rheological, and morphological characteristics, for this 15% RP blend.

### 3.2. Effect of Multiple Recycling Cycles (15% RP Blend)

#### 3.2.1. Mechanical Properties

The influence of up to 10 recycling cycles on the tensile and flexural properties of the 15% RP blend was investigated.

**Figure 3:**
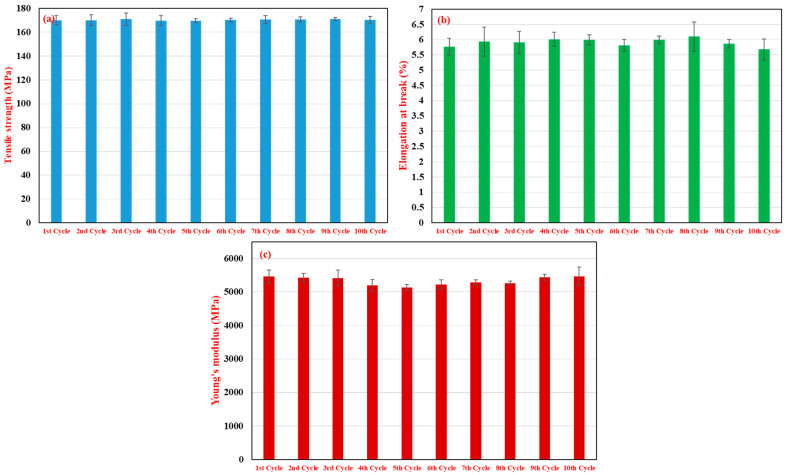
Effect of multiple recycling cycles (1–10 cycles, 15 wt.% RP blend) on the tensile properties of 35% glass fiber-reinforced PA66 composites: (**a**) tensile strength (MPa), (**b**) elongation at break (%), and (**c**) Young’s modulus (MPa).

Tensile properties, including tensile strength, elongation at break, and Young’s modulus, are presented in Figure 3. As discussed previously, tensile strength (Figure 3a) remained remarkably stable, fluctuating narrowly around 170 MPa (e.g., 170.10 MPa for first cycle, 169.62 MPa for fifth cycle, and 170.36 MPa for tenth cycle). This stability suggests that the cumulative effects of degradation and fiber attrition do not severely compromise the tensile strength within 10 reprocessing cycles for this specific 15% RP blend. Elongation at break (Figure 3b) and Young’s modulus (Figure 3c) also showed relative consistency, with minor variations likely falling within experimental error.

**Figure 4:**
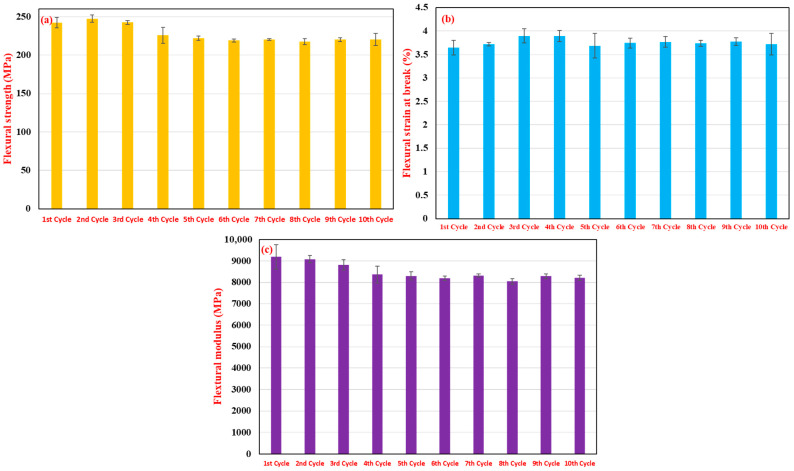
Effect of multiple recycling cycles (1–10 cycles, 15 wt.% RP blend) on the flexural properties of 35% glass fiber-reinforced PA66 composites: (**a**) flexural strength (MPa), (**b**) flexural strain at break (%), and (**c**) flexural modulus (MPa).

The flexural properties of the 15% RP blend across the 10 recycling cycles are illustrated in Figure 4. Flexural strength, as shown in Figure 4a, exhibited a discernible decreasing trend with an increasing number of recycling cycles. It started at approximately 242.21 ± 6.71 MPa for the first cycle and gradually decreased to around 220.21 ± 7.92 MPa by the tenth cycle. This represents a reduction of approximately 9% over 10 cycles. Similarly, the maximum flexural strain (or flexural strain at break), depicted in Figure 4b, showed a slight, but generally decreasing trend, starting at 3.65 ± 0.15% for the first cycle and reducing to 3.72 ± 0.23% (with some fluctuations, e.g., 3.90% at third and fourth cycles, then decreasing) by the tenth cycle. The flexural modulus, presented in Figure 4c, also demonstrated a clear gradual decline with more reprocessing. The modulus was approximately 9192.08 ± 569.17 MPa after the first cycle, decreasing to around 8215.17 ± 123.52 MPa after the tenth cycle, indicating a reduction in stiffness of about 10.6%. This progressive reduction in flexural strength and modulus with repeated recycling is likely attributable to the cumulative effects of fiber attrition, leading to shorter fibers that are less effective in resisting bending loads, and potential degradation of the polymer matrix reducing its ability to transfer stress efficiently to the fibers [17,35,36].

**Figure 5:**
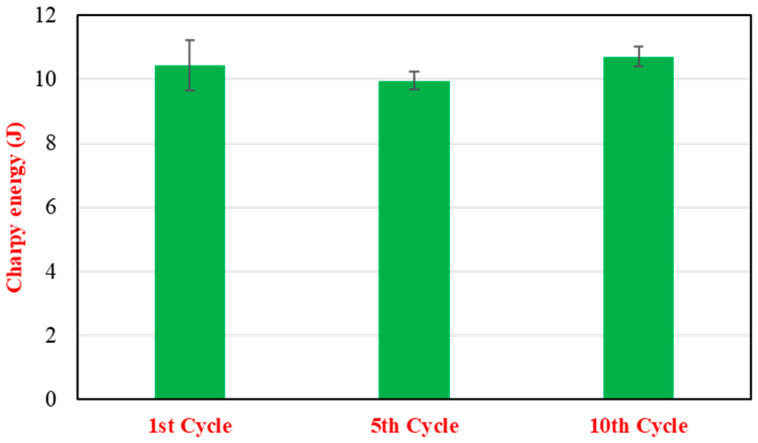
Charpy impact energy (J) of 35% glass fiber-reinforced PA66 composites (15 wt.% RP blend) as a function of recycling cycles (1–10).

The Charpy impact energy of the 15% RP blend as a function of recycling cycles is depicted in Figure 5. The impact energy remained relatively constant across the 10 cycles. Specifically, the first cycle exhibited an impact energy of 10.44 ± 0.77 J. After five cycles, the value was 9.96 ± 0.28 J, and by the tenth cycle, it had recovered slightly to 10.71 ± 0.31 J. These minor fluctuations suggest no significant degradation in the material’s toughness or its ability to absorb impact energy over multiple reprocessing steps, indicating good retention of this critical property. The observed resilience in tensile and impact properties after multiple cycles is a positive indicator for in-process recycling, though the more pronounced decline in flexural properties warrants careful consideration for applications where bending stiffness and strength are critical design parameters.

#### 3.2.2. Thermal Properties and MFI

**Figure 6:**
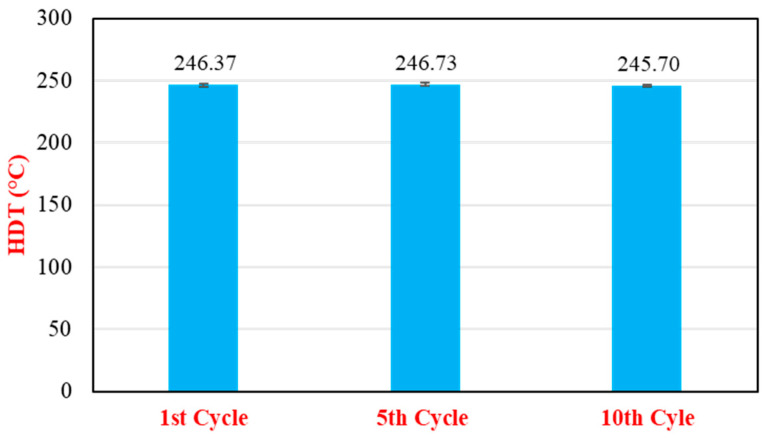
Heat deflection temperature (HDT) of %35GF-PA66 composites (15% RP blend) as a function of the number of recycling cycles.

HDT values (Figure 6) remained remarkably stable across the 10 recycling generations, fluctuating within a narrow range of approximately 1 °C. The values were 246.37 ± 1.44 °C (first cycle), 246.73 ± 1.43 °C (fifth cycle), and 245.70 ± 1.13 °C (tenth cycle). This high level of stability signifies that the cumulative effects of reprocessing had a negligible impact on the material’s short-term thermomechanical performance under load. This suggests that any polymer degradation or fiber shortening occurring during recycling was not severe enough to significantly alter the HDT of the composite [20,37].

**Figure 7:**
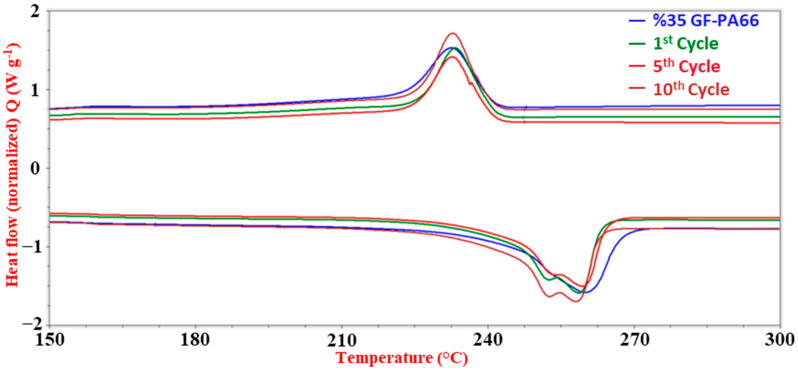
DSC thermograms of 35% glass fiber-reinforced PA66 composites (15 wt.% RP blend) for virgin material and after 1, 5, and 10 recycling cycles, showing melting and crystallization behavior.

DSC analysis (Table 2 and Figure 7) revealed that T_m_ (around 252–259 °C) and T_c_ (around 232–233 °C) remained largely unaffected by the number of recycling cycles, suggesting that the bulk crystalline structure and its melting and crystallization behavior were preserved.

**Figure 8:**
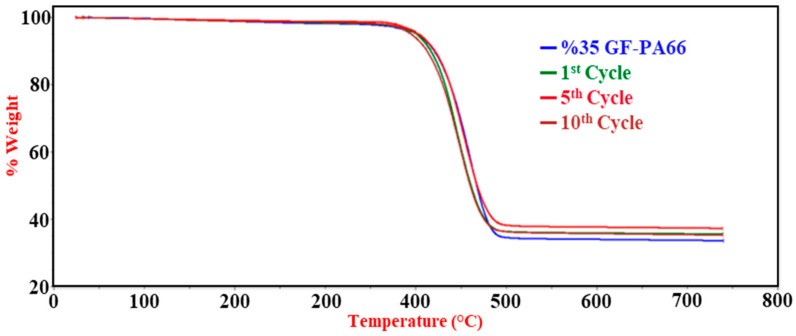
TGA of 35% glass fiber-reinforced PA66 composites (15 wt.% RP blend) for virgin material and after 1, 5, and 10 recycling cycles, showing thermal degradation profiles.

**Figure 9:**
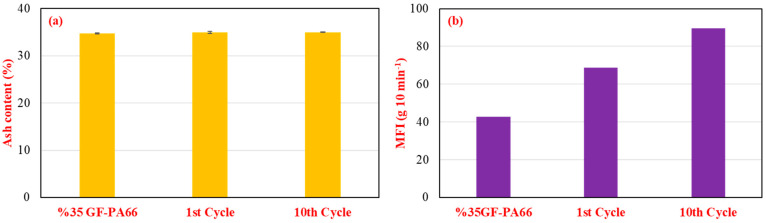
Effect of multiple recycling cycles (1–10 cycles, 15 wt.% RP blend) on 35% glass fiber-reinforced PA66 composites: (**a**) ash content (wt.%), indicating glass fiber retention, and (**b**) melt flow index (g/10 min), reflecting molecular weight changes.

TGA results (Table 3 and Figure 8) showed similar single-step degradation profiles across all cycles. The maximum degradation temperature (T_max_) exhibited a slight decrease with more cycles. Ash content (Figure 9a) marginally increased from 34.72% (virgin equivalent) to 35.01% after 10 cycles, possibly reflecting a slight preferential degradation of the polymer matrix compared to the inert glass fibers over repeated thermal exposures. The MFI values (Figure 9b) demonstrated a pronounced increase with successive recycling cycles, rising from approximately 40 g/10 min (first cycle) to around 85 g/10 min (tenth cycle). This substantial MFI increase is a strong indicator of significant chain scission and a reduction in the average molecular weight of the PA66 matrix stemming from the cumulative thermomechanical shear and thermal history during repeated processing [19,38,39].

### 3.3. Morphological Analysis (SEM)

**Figure 10:**
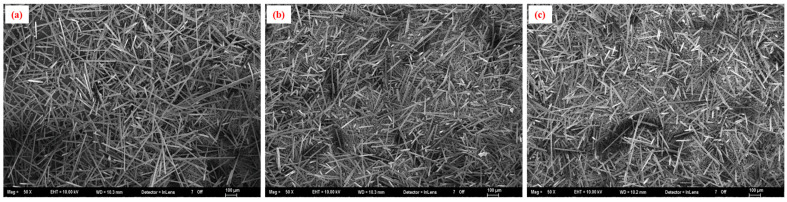
SEM micrographs of ash residues from 35% glass fiber-reinforced PA66 composites: (**a**) virgin material, (**b**) after 1 recycling cycle (15 wt.% RP), and (**c**) after 10 recycling cycles (15 wt.% RP), showing qualitative changes in fiber morphology. Scale bars: [100 µm].

**Figure 11:**
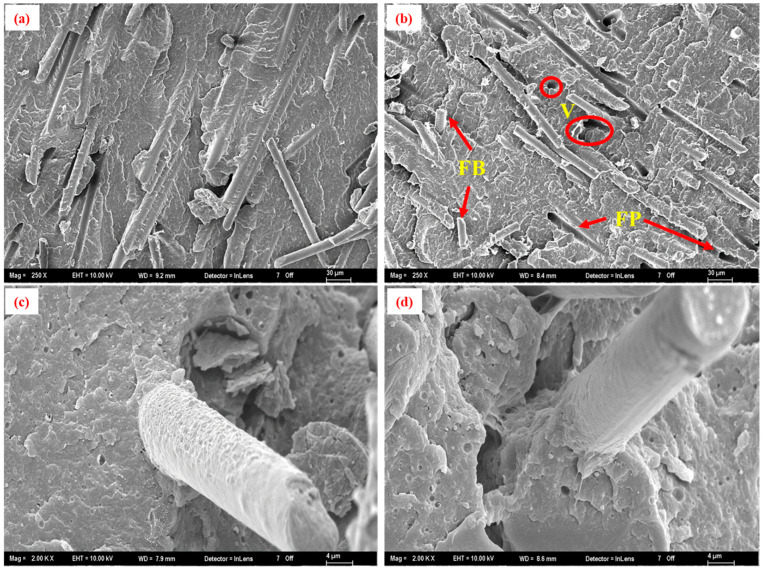
SEM micrographs of tensile fracture surfaces of 35% glass fiber-reinforced PA66 composites (15 wt.% RP blend): (**a**,**c**) after 1 recycling cycle, showing fiber pullout and matrix adhesion; (**b**,**d**) after 10 recycling cycles, revealing increased void formation and shorter pulled-out fibers. Arrows indicate instances of fiber pullout (FP), fiber breakage (FB), and voids at the fiber–matrix interface (V). Micrographs (**c**,**d**) are higher magnifications of the regions in (**a**,**b**), respectively.

SEM micrographs of the ash residues from virgin %35GF-PA66 and samples after 1 and 10 recycling cycles (Figure 10) visually indicated a qualitative reduction in average fiber length and a broader distribution of fiber lengths with increased recycling, although quantitative fiber length distribution analysis was not performed in this study. Fracture surfaces of tensile specimens from the first and tenth recycling cycles (Figure 11) revealed features characteristic of fiber-reinforced composite failure, including fiber pullout, fiber breakage, and matrix deformation. After 10 recycling cycles (Figure 11b–d), there appeared to be an increase in the prevalence of voids at the fiber–matrix interface and more instances of shorter pulled-out fibers compared to the first cycle (Figure 11a–c). This could suggest some degradation of fiber–matrix adhesion or increased matrix brittleness due to polymer degradation [40,41,42,43]. The shorter fibers and potentially weakened interface contribute to the observed trends in mechanical properties, particularly the decrease in flexural stiffness.

### 3.4. Overall Discussion

These comprehensive results demonstrate that mechanical recycling of %35GF-PA66 runner waste for in-process reuse is a technically promising strategy. The incorporation of up to 15% RP or subjecting a 15% RP blend to up to 10 reprocessing cycles led to relatively modest changes in key mechanical properties, particularly tensile strength and impact resistance. The most sensitive indicator of degradation was the MFI, which increased significantly, pointing to a reduction in polymer molecular weight. This is a common consequence of thermomechanical degradation during melt processing [38,39]. The slight decrease in HDT and flexural properties also aligns with this degradation and the inevitable fiber attrition.

From an industrial perspective, the stability of tensile strength and impact energy, which are often critical design parameters for components like injector sockets, is particularly encouraging. The observed changes are well within acceptable tolerance limits for many demanding applications. This finding is particularly impactful when considering the specific manufacturing context of this study. The runner system for the diesel injector socket mold used generates waste equivalent to 13% of the total shot weight. Our decision to conduct the extensive 10-cycle reprocessing study on a 15% RP blend was therefore a deliberate choice to validate a recycling strategy that is not only capable of reintegrating 100% of the generated runner waste but also provides a practical operational buffer. The excellent retention of key mechanical properties at this 15% RP level demonstrates that a robust, closed-loop, in-process recycling system is technically feasible.

This provides a direct, data-driven pathway for manufacturers to eliminate this waste stream entirely, leading to significant cost savings and environmental benefits without compromising component quality for this application [11,12]. However, for applications with extremely tight dimensional tolerances or those highly sensitive to melt viscosity variations, the increase in MFI would need careful management in process control. Future optimization could involve less aggressive grinding techniques or the incorporation of stabilizers to mitigate degradation during reprocessing [44]. Ultimately, the findings strongly support the move towards a more circular economy within the automotive component manufacturing sector.

## 4. Conclusions

This study systematically evaluated the effects of incorporating mechanically recycled %35GF-PA66 runner waste and multiple reprocessing cycles on the material’s properties. The key conclusions are:

1. The incorporation of up to 15% recycled %35GF-PA66 runner waste into virgin material resulted in only minor reductions in tensile and flexural strength, while elongation at break tended to increase slightly.
2. Multiple reprocessing cycles (up to 10 cycles with a 15% RP blend) demonstrated good retention of tensile strength and Charpy impact energy. HDT remained remarkably stable (variation of ~0.7 °C after 10 cycles), while flexural properties exhibited a more noticeable gradual decline.
3. The melt flow index (MFI) increased significantly with successive recycling cycles, confirming thermomechanical degradation and a reduction in the average molecular weight of the PA66 matrix.
4. DSC analysis indicated that the primary melting and crystallization temperatures were largely unaffected by the recycling processes, suggesting the bulk crystalline structure remained stable.
5. SEM analysis provided qualitative evidence of fiber attrition and increased void formation at the fiber–matrix interface with more extensive recycling.
6. Overall, the in-process mechanical recycling of %35GF-PA66 runners is a technically viable option for applications such as diesel injector sockets, particularly when recycled content is managed (e.g., up to 15%) and the number of reprocessing generations is considered. The observed property changes are generally modest and offer a pathway to significant cost savings and improved manufacturing sustainability.

Further research focusing on quantitative fiber length distribution, the effect of recycling on long-term performance (fatigue, creep), and the potential use of process stabilizers or novel compatibilizers to enhance the properties of blends with higher recycled content could further optimize the reuse of this valuable material stream.

## Figures and Tables

**Table 1:** Injection molding process parameters.

Parameter	Value	Parameter	Value
Injection Speed	70 mm/s	Nozzle Temperature	260 °C
Screw Speed	40%	Zone 1 Temperature	275 °C
Shot Size	75 mm	Zone 2 Temperature	270 °C
Holding Pressure Time	10 s	Zone 3 Temperature	265 °C
Cooling Time	15 s	Feed Zone Temperature	30 °C
Mold Temperature	80 °C	Back Pressure	5 bar

**Table 2:** Key DSC thermal properties (melting temperature T_m_, crystallization temperature T_c_, and associated enthalpies) of %35GF-PA66 composites (15% RP blend) as a function of the number of recycling cycles.

Parameter	T_c_ (°C)	ΔH_c_ (J/g)	T_m_ (°C)	ΔH_m_ (J/g)
Virgin (%35GF-PA66)	232.57	33.83	259.89	43.15
1st Cycle	233.19	35.07	252.58	45.05
			258.71	
5th Cycle	232.68	33.62	253.78	44.08
			259.41	
10th Cycle	232.76	36.3	252.71	47.92
			258.19	

**Table 3:** Summary of TGA results (onset degradation temperature, maximum degradation temperature, weight loss percentages) for %35GF-PA66 composites (15% RP blend) as a function of the number of recycling cycles.

Recycling Cycle	T_max-degrad_ (°C) (N_2_)	Weight Loss (%) (N_2_, ~350–500 °C)	Residual Ash (%) (at 900 °C, O_2_)
Virgin (%35GF-PA66)	459.88	66.34	33.67
1st Cycle	444.56	64.42	35.82
5th Cycle	452.89	62.69	37.31
10th Cycle	447.57	64.72	35.28

## Data Availability

The data that support the findings of this study are available from the corresponding author upon reasonable request.

## Abbreviations

The following abbreviations are used in this manuscript.

%35GF-PA66	35% glass fiber-reinforced Polyamide 6,6
PA66-GF	Polyamide 6,6 reinforced with short glass fibers
RP	recycled polymer
HDT	heat deflection temperature
MFI	melt flow index
DSC	differential scanning calorimetry
TGA	thermogravimetric analysis
SEM	scanning electron microscope
FRPs	fiber-reinforced polymers
